# Raman Spectroscopic Analysis of the Reaction between Al-Si Coatings and Steel

**DOI:** 10.1021/acsomega.3c01938

**Published:** 2023-07-17

**Authors:** Jixi Zhang, Kyle J. Daun, Rodney D. L. Smith

**Affiliations:** † Department of Chemistry, University of Waterloo, 200 University Avenue W., Waterloo, Ontario N2L 3G1, Canada; ‡ Department of Mechanical and Mechatronics Engineering, University of Waterloo, 200 University Avenue W., Waterloo, Ontario N2L 3G1, Canada; § Waterloo Institute for Nanotechnology, University of Waterloo, 200 University Avenue W., Waterloo, Ontario N2L 3G1, Canada

## Abstract

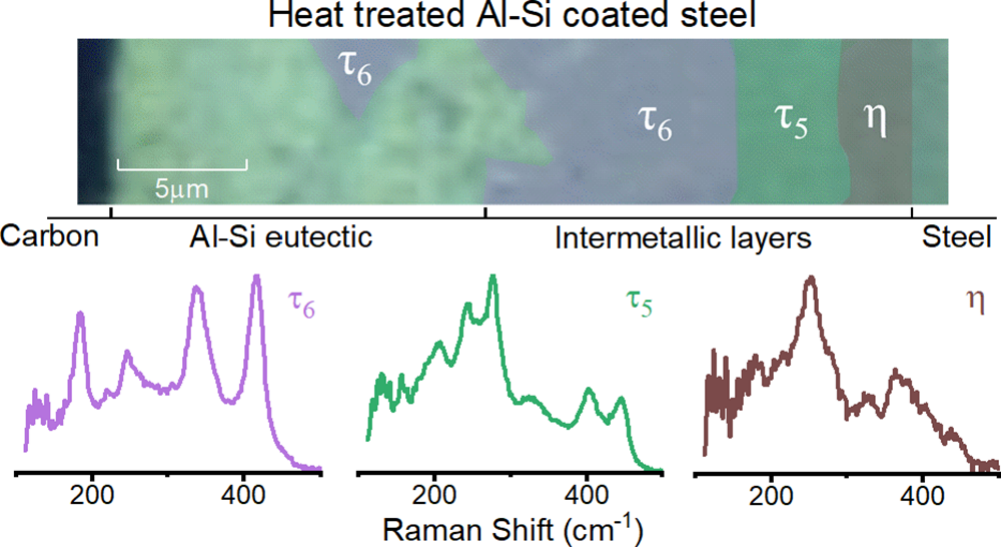

Hot-stamped ultrahigh strength steel components are pivotal to automotive light-weighting. Steel blanks, often coated with an aluminum-silicon (Al-Si) layer to protect them from oxidation and decarburization, are austenitized within a furnace and then simultaneously quenched and formed into shape. The Al-Si coating melts within the furnace and reacts with iron from the steel to yield an intermetallic phase that provides some long-term corrosion protection. During the intermediate liquid phase, some of the coating may transfer to the furnace components, leading to maintenance costs and operational downtime. A detailed understanding of the coating transformation mechanism is needed to avoid such production issues while ensuring that final intermetallic coatings conform to specifications. We introduce cross-sectional Raman microscopic mapping as a method to rapidly elucidate the coating transformation mechanism. Raman spectroscopic fingerprints for relevant intermetallic compounds were determined using synthesized Al-Fe-Si ternary and Al-Fe binary compounds. These fingerprints were used to map the spatial distribution of intermetallic compounds through cross sections of Al-Si-coated 22MnB5 specimens that were heated at temperatures between 570 and 900 °C. These chemical maps show that the intermetallic fraction of the coating does not grow significantly until formation of η (Al_5_Fe_2_) at the steel interface, suggesting that η facilitates extraction of iron from the steel and subsequent diffusion through the coating. Under the heating conditions used here, a series of reactions ultimately lead to a silicon-rich τ_2_ (Al_3_FeSi) phase on top of the binary η phase. The technique presented here simplifies structural analysis of intermetallic compounds, which will facilitate prototyping of strategies to optimize hot stamping.

## Introduction

Hot stamping is used widely to manufacture ultrahigh strength steel automotive parts.^1−4^ In this process, steel blanks austenitized, most often within a roller hearth furnace, before they are simultaneously quenched and shaped within a cooled die.^1,5−7^ The steel blanks are usually equipped with a 90% Al/10% Si (wt) coating to prevent oxidation and decarburization within the furnace.^4,8−10^ The aluminum-silicon (Al-Si) coating melts at ca. 577 °C^8,11−14^ and undergoes a reaction with Fe from the steel substrate, yielding an Al-Fe-Si intermetallic coating that provides long-term corrosion resistance to the automotive parts.^4,15^ Unfortunately, transfer of the intermediate liquid phase to ceramic rollers in the furnace leads to destruction of the rollers, among other production issues.^16^ A reliable understanding of the transport and kinetic processes underlying transformation of the Al-Si coating into the Al-Fe-Si intermetallic layer is crucial in designing strategies to alleviate coating transfer while ensuring that the final coating conforms to specification.

The Al-Si coating undergoes a series of complex liquefaction-solidification reactions associated with the transfer of iron from the steel substrate to the coating.^5,17,18^ Efforts to identify the chemical reaction steps associated with the overall reaction have largely focused on ex situ electron microscopic analysis of specimens heated within furnaces at different temperatures.^8,13,14,17,19−22^ As-received samples typically consist of an Al-Si matrix atop a thin intermetallic layer at the steel-coating interface. The intermetallic interface forms during the dip-coating process, with τ_5_ (Al_7_Fe_2_Si) and τ_6_ (Al_4.5_FeSi) being the most commonly reported components.^4,5,8,9,18^ Electron microscopy, energy-dispersive X-ray spectroscopy (EDS), and electron back-scattering diffraction have been used to identify a range of intermetallic compounds that emerge during the coating transformation process, including the ternary Al-Fe-Si compounds commonly denoted τ_1_ through τ_6_, and the binary phases η (Al_5_Fe_2_), θ (Al_13_Fe_4_), α_2_ (AlFe), and ζ (Al_2_Fe).^4,8,17,19,20^ The aluminum-rich corner of the ternary Al-Fe-Si phase diagram is complex, with numerous invariant reactions linking together three or more phases.^21,23,24^ The gradual conversion of the Al-Si coating to a complete Al-Fe-Si intermetallic coating requires an increase in the Fe content of the coating, which means that the coating composition must change with time and temperature. This change in coating composition introduces the possibility of partial solidification and of localized interface-specific reactions. This, in turn, raises questions regarding the mechanistic and kinetic importance of individual phases formed within the coating as the structure evolves. In situ light reflection measurements made by shining a laser onto the surface of a heated specimen indicate that a multistep liquefaction-resolidification process occurs between ca. 570 and 700 °C,^9^ with coating thickness affecting times required for complete conversion of the coating.^25^

Raman microscopy is frequently used to characterize solid-state materials, but it has seen little application in the analysis of Al-Si coatings and Al-Si-Fe intermetallics. Raman microscopy on solids can be challenging due to inherently weak signals and the possibility of fluorescence, but measurement of vibrational frequencies of the lattice provides a chemical fingerprint that enables speciation with greater confidence than techniques such as EDS.^26−30^ Klassen et al. recently pioneered the application of Raman microscopy to Al-Si-coated steels under in situ high-temperature conditions.^18^ The analysis identified several unique spectra that emerged at temperature thresholds. These spectra were assigned based on ex situ analysis of cooled specimens by EDS and comparison with previously published reaction mechanisms. This work demonstrated Raman microscopy to be a powerful technique capable of detecting intermetallic compounds formed in the Al-Fe-Si system. The surveyed sampling approach, however, limits mechanistic information that could be extracted, and the lack of crystalline standards for comparisons makes the Raman spectrum assignments tentative in nature. A systematic spectroscopic analysis of cross sections of the thin coatings will more comprehensively capture the spatial distribution of phases throughout the coating. When expanded across varied temperatures, such a systematic approach has the potential to provide broader insights into the mechanism by which the structure of the coatings evolves.

Herein, we introduce cross-sectional Raman microscopic mapping as a new strategy to study the mechanism by which Al-SI coatings are converted to an intermetallic coating on steel substrates. Intermetallic phases were synthesized and characterized by Raman spectroscopy and X-ray diffraction (XRD) measurements to firmly establish the Raman vibrational fingerprints for relevant phases. Comparison of the Raman spectra acquired on a series of Al-Si-coated steel sheets heated at temperatures between 570 and 900 °C with these fingerprints enables identification of the temperature-dependent composition of the Al-Si coating. The spatial information provided using this spectroscopic mapping protocol shows that the coating structure evolves as a series of interface-specific chemical reactions, with τ_5_, η, and τ_2_ playing important roles in the overall transformation.

## Experimental Section

### Synthesis

A series of binary Al-Fe and ternary Al-Fe-Si phases were synthesized for use as characterization standards by combining appropriate molar ratios of pure Al granules (Puratronic, 99.999%), Fe powder (Alfa Aesar, 98%), and Si powder (Alfa Aesar, 99.9%) and melting them together using an electric arc under an argon atmosphere.^31^ The ingot obtained upon cooling was crushed using an iron mortar and pestle and then subsequently ground to a powder using an agate mortar and pestle. Targeted samples include τ_2_ (Al_3_FeSi), τ_3_ (Al_2_FeSi), τ_4_ (Al_3_FeSi_2_), τ_5_ (Al_7_Fe_2_Si), τ_6_ (Al_9_Fe_2_Si_2_), η (Al_5_Fe_2_), and θ (Al_13_Fe_4_).

Furnace-heated specimens were prepared using Al-Si-coated 22MnB5 steel from a commercial supplier (AS150 Usibor 1500, ArcelorMittal). Manufacturer specifications indicate that the composition of the steel includes a maximum of 0.25 wt % C, 0.4 wt % Si, 0.03 wt % P, 0.01 wt % S, 0.1 wt % Al, 1.4 wt % Mn, 0.35 wt % Cr, 0.05 wt % Ti, 0.2 wt % Cu, 0.01 wt % Nb, and 0.005 wt % B, with the balance being Fe.^32^ The Al-Si coating has a nominal composition of 10% (wt) Si and a weight of 1.28 g/cm^2^, corresponding to an as-received thickness of approximately 25 μm. The specimens were cut into 38 mm × 19 mm pieces and degreased and cleaned with ethanol and then deionized water. A total of 12 cleaned specimens were heated for 10 min in a muffle furnace at a set-point temperature and then removed from the furnace and allowed to passively cool to room temperature. Set points of 570, 580, 600, 610, 620, 630, 640, 650, 660, 670, 800, and 900 °C were selected to capture temperature ranges where significant structural evolution has been previously noted.^9,18,25^ Cross sections of each heated sample, and an as-received sample, were mounted and polished for further characterization.

### Structural Characterization

Powder XRD was performed on the characterization standards using either an INEL X-ray diffractometer (τ_2_, τ_3_, τ_5_, τ_6_, and θ) or a PANalytical Empyrean X-ray diffractometer (τ_4_ and η). Diffraction patterns were compared with previously published structures found in the Inorganic Crystal Structure Database (ICSD).^33^ Rietveld refinements were performed using the GSAS-II software package.^34^

Raman microscopy measurements were performed using a Renishaw inVia Reflex system equipped with a sample stage capable of 100 nm positioning in three dimensions. A 532 nm (Renishaw DPSSL laser, 50 mW) laser filtered to 10% intensity was focused on the samples using a 50× objective, with a 2400 lines/mm grating used to analyze Raman scattered light. This provided a Raman shift spacing of 1.2 cm^–1^ between individual data points and a maximum spatial resolution of approximately 650 nm. Spectra on characterization standards were acquired between 111 and 1370 cm^–1^ with a total acquisition time of 20 s. The surface area of the powders was surveyed to ensure that the representative spectra were obtained. Spectra on heat-treated steel plates were acquired across using a 2-D grid with 1 μm spacing between spectra acquired in the *x* and *y* dimensions with the same acquisition settings used on measurement standards. Grid size is variable per sample but typically on the order of 5 μm by 30 μm. All spectra were acquired and analyzed using the Renishaw 5.5 software package. Spectra were prepared for analysis by simple normalization of the data between 111 and 500 cm^–1^ to span zero to unity.

## Results

### Characterization Standards

**Figure 1:**
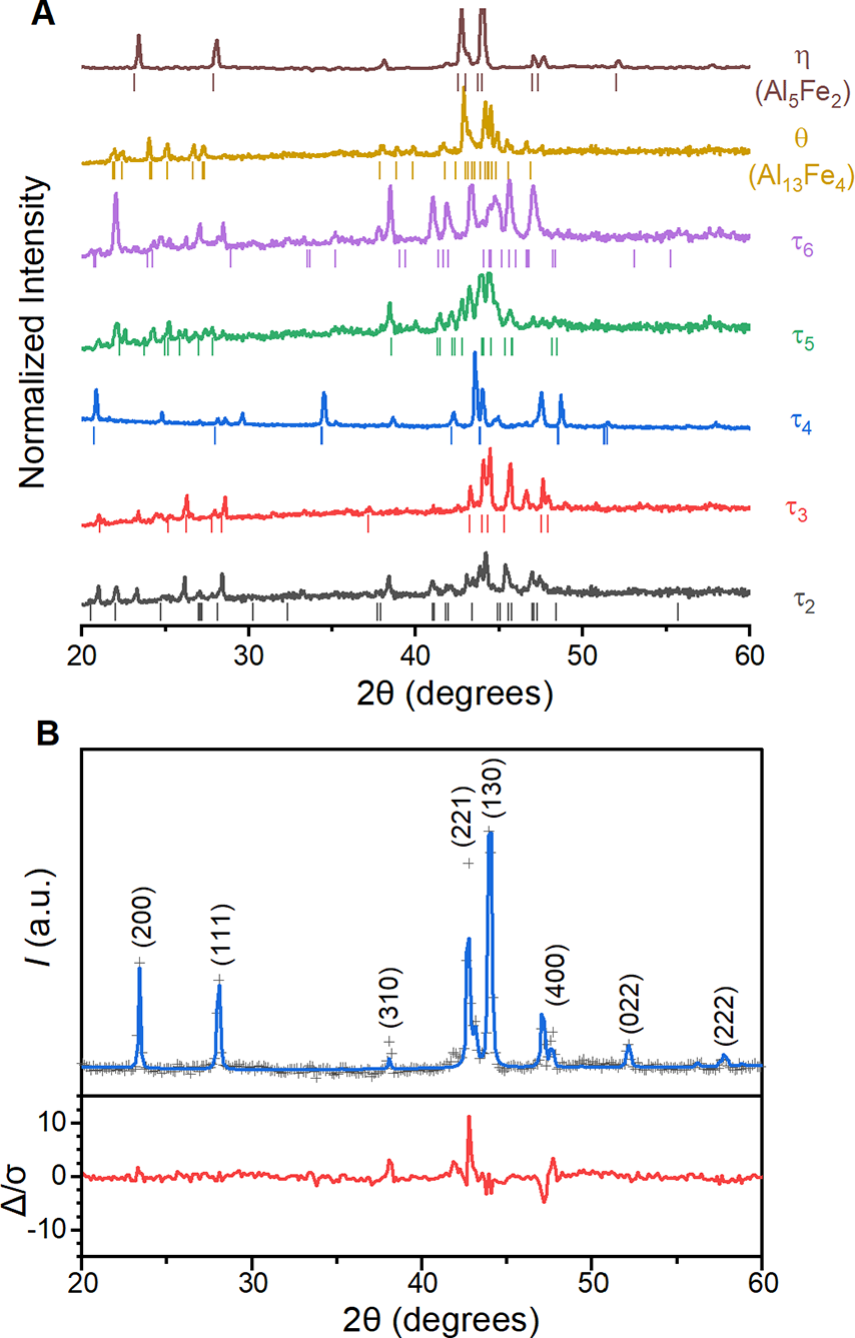
Powder XRD patterns for synthesized intermetallic phases. (A) Experimental patterns (solid lines) compared to Bragg peaks predicted by ICSD structures cited in the text (vertical dashes). (B) Sample Rietveld refinement performed on synthesized η. The remaining samples are provided in Figures S1–S6.

A series of synthesized intermetallic standards were characterized to identify the Raman spectra for structures of interest. Powder XRD patterns indicate that a single phase is present for the binary compositions θ (Al_13_Fe_4_) and η (Al_5_Fe_2_) but contaminants in all ternary phases (τ_2_–τ_6_; Figure 1A). Such impurities in the ternary phases are to be expected, given the complex interaction between many phases in the aluminum-rich corner of the ternary phase diagram for Al-Fe-Si.^21,23,24^ Rietveld refinements on each XRD pattern nonetheless confirm that the majority component of each characterization standard is the desired phase: θ can be refined to the *C*2/*m* space group (ICSD 57795),^35^ η to *Cmcm* (ICSD 57796),^36^ τ_2_ to *R*-3 (ICSD 99169),^37^ τ_3_ to *Cmma* (ICSD 40317),^38^ τ_4_ to *I*4/*mcm* (ICSD 199347),^39^ and τ_5_ to *P*6_3_/*mmc* (ICSD 422224).^40^ Refinements on attempted syntheses of τ_6_ showed a small amount of the expected *A*2/*a* (ICSD #54050) phase (Figure 1B and Figures S1–S6)^41^ but always as a minor component. Metallic Al was found in τ_2_, τ_4_, τ_5_ and τ_6_, seen as Bragg peaks that match those expected for the *Fm*-3*m* space group (ICSD 18839).^42^ These characterization standards provide sufficient purity for the purposes of Raman microscopic characterization.

**Figure 2:**
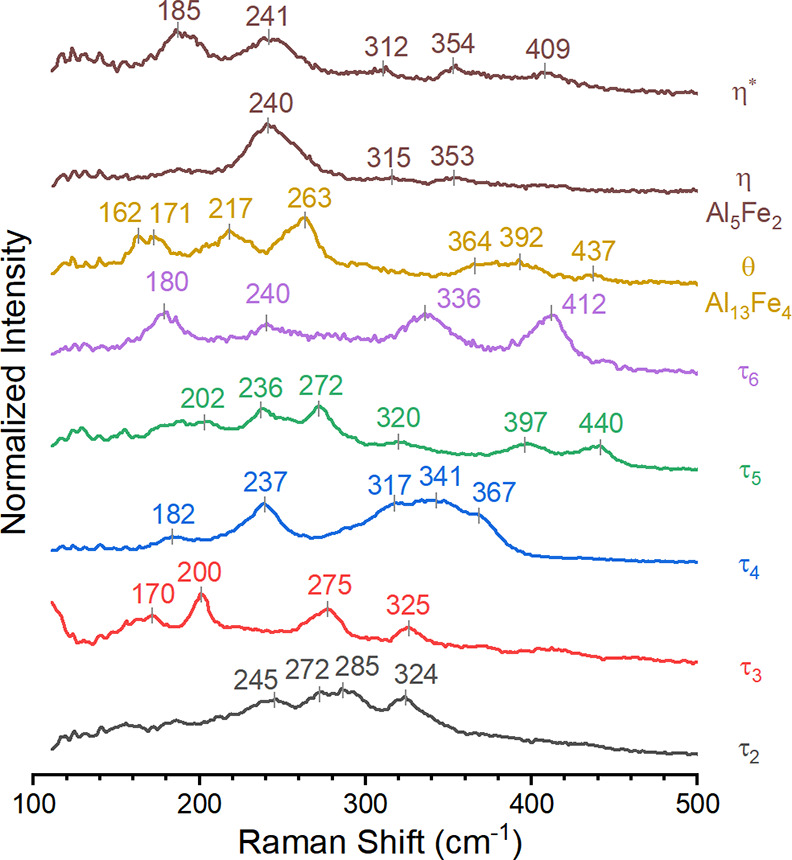
Raman spectra assigned to each phase using characterization standards.

Spectroscopic analysis of the characterization standards with a Raman microscope identified the major components and impurities. A series of at least 15 Raman spectra were acquired across the surface of each characterization standard. Spectra that were mutually consistent for each characterization standard were averaged together to enhance the signal-to-noise ratio. These spectra show distinctive vibrational fingerprints in the region between 100 and 500 cm^–1^; the Raman spectrum for the Al-Si mixture was consistent with that of elemental silicon, with a single strong peak at ca. 520 cm^–1^. The assignment of each individual spectrum to a given intermetallic phase (Figure 2) was performed by considering the prevalence of each type of the Raman spectrum for a given standard. Assignments were then cross-comparing with the spectra acquired on the other standards. This approach confirmed pure phases of θ and η, albeit with η showing variations in intensity of peaks that may indicate strain or lattice defects. Ternary samples contained the phase of interest plus contaminant phases: τ_2_ contained contributions from τ_6_, τ_3_ contained τ_2_, τ_4_ contained τ_3_, and τ_5_ contained τ_2_ and θ. The purity of τ_6_ is lowest for all synthesized samples, as seen with XRD, yielding five unique Raman spectra within the samples (Figure S7). Three of these spectra can be assigned as τ_2_, τ_4_, and θ. One of the remaining two spectra is tentatively assigned as τ_6_ based on the emergence of this spectrum in the coating at temperatures expected for τ_6_ and on previous assignment.^18^ Spectrum assignments for τ_5_, τ_6_, η, and θ agree with past assignments.^18^ To the best of our knowledge, the remaining spectra have not been previously reported.

#### Structural Evolution of Coatings

**Figure 3:**
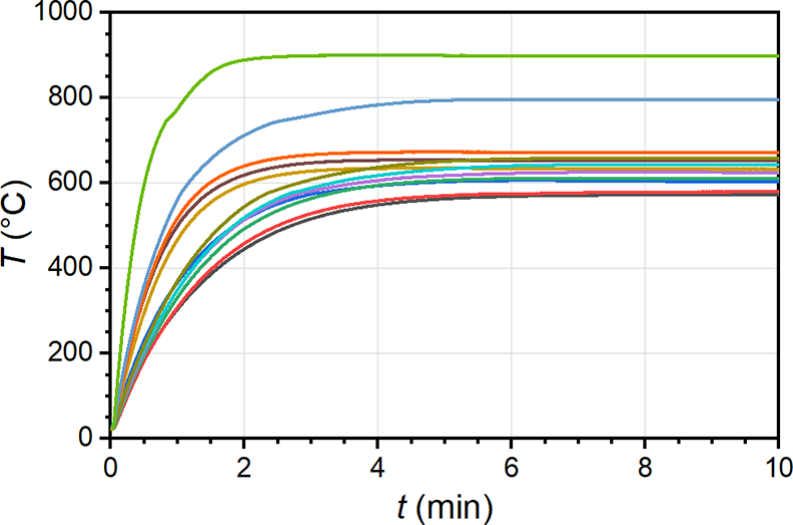
Temperature profiles acquired on Al-Si-coated 22MnB5 steel coupons placed in a tube furnace. Set-point temperatures are in 10 °C steps from 570 to 670 °C, plus 800 and 900 °C.

The time required for Al-Si-coated 22MnB5 steel coupons to attain a stable temperature was determined by measuring temperature as a function of time. Significant structural evolution of the Al-Si coatings is known to occur between 570 and 670 °C.^5,9,25^ A furnace was preheated to each selected set-point temperature before placing a steel sample with an attached thermocouple within the center of the furnace. Temporal temperature measurements indicate that the steel samples reach the furnace set-point temperature between 5 and 7 min, depending on the set-point temperature (Figure 3). While both time and temperature variables are important to completely describe the evolution of the coating structure, a single heating period of 10 min was used for all samples to establish the viability of cross-sectional spectroscopic analysis.

**Figure 4:**
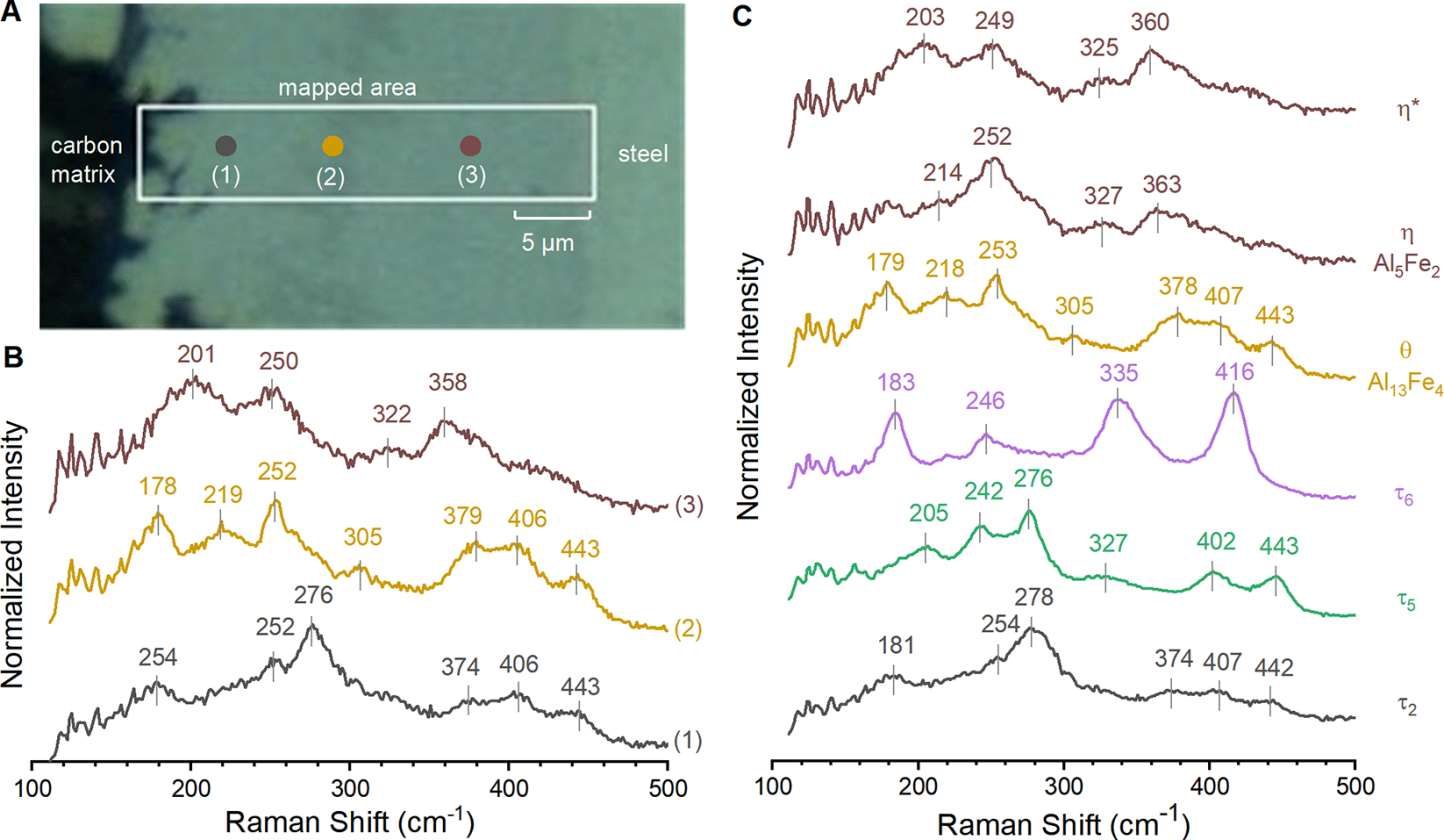
Cross-sectional Raman microscopic mapping of heat-treated Al-Si-coated 22MnB5 steel coupons. (A) White light microscope image of the mapped area, with a box around the mapped area. The sample shown corresponds to heat treatment at 800 °C for 10 min. (B) Raman spectra acquired at the positions in the white light image indicated. (C) All unique spectra identified across all 4098 spectra in 13 cross-sectional Raman microscopic maps on temperature-treated samples.

Cross-sectional Raman microscopic mapping was used to identify intermetallic phases present in Al-Si-coated 22MnB5 after heating at temperature set points. Cooled specimens were mounted within carbon pucks and polished to a mirror finish to expose a cross section of the steel coupon and the coating. Raman microscopic mapping was performed by acquiring discrete spectra in 1 μm steps to generate a 2-D map. Map sizes vary slightly due to variations in the thickness of the Al-Si coatings, but each map was at least 26 μm by 6 μm. These maps extend from the top of the Al-Si coating to the steel interface. A sample of the mapping procedure shows the area mapped for the sample heated at 800 °C (Figure 4A) and the three unique spectra observed within this sample (Figure 4B). This treatment at 800 °C yields a layered structure, where a continuous layer of η sits atop the steel substrate and is followed by sequential layers of θ and τ_2_. Inspection of all 4098 spectra obtained across the 13 Raman microscopic maps identified a total of 6 unique spectra that are presented as an average spectrum on a per-phase and per-sample basis (Figure S8) and as the overall average across all samples on a per-phase basis (Figure 4C). In addition, spectra for the Al-Si mixture and elemental silicon were observed as a single, strong peak at ca. 520 cm^–1^. Comparison of these unique spectra to the characterization standards shows that the dominant components of these heat-treated samples are τ_2_, τ_5_, τ_6_, θ, and η.

**Figure 5:**
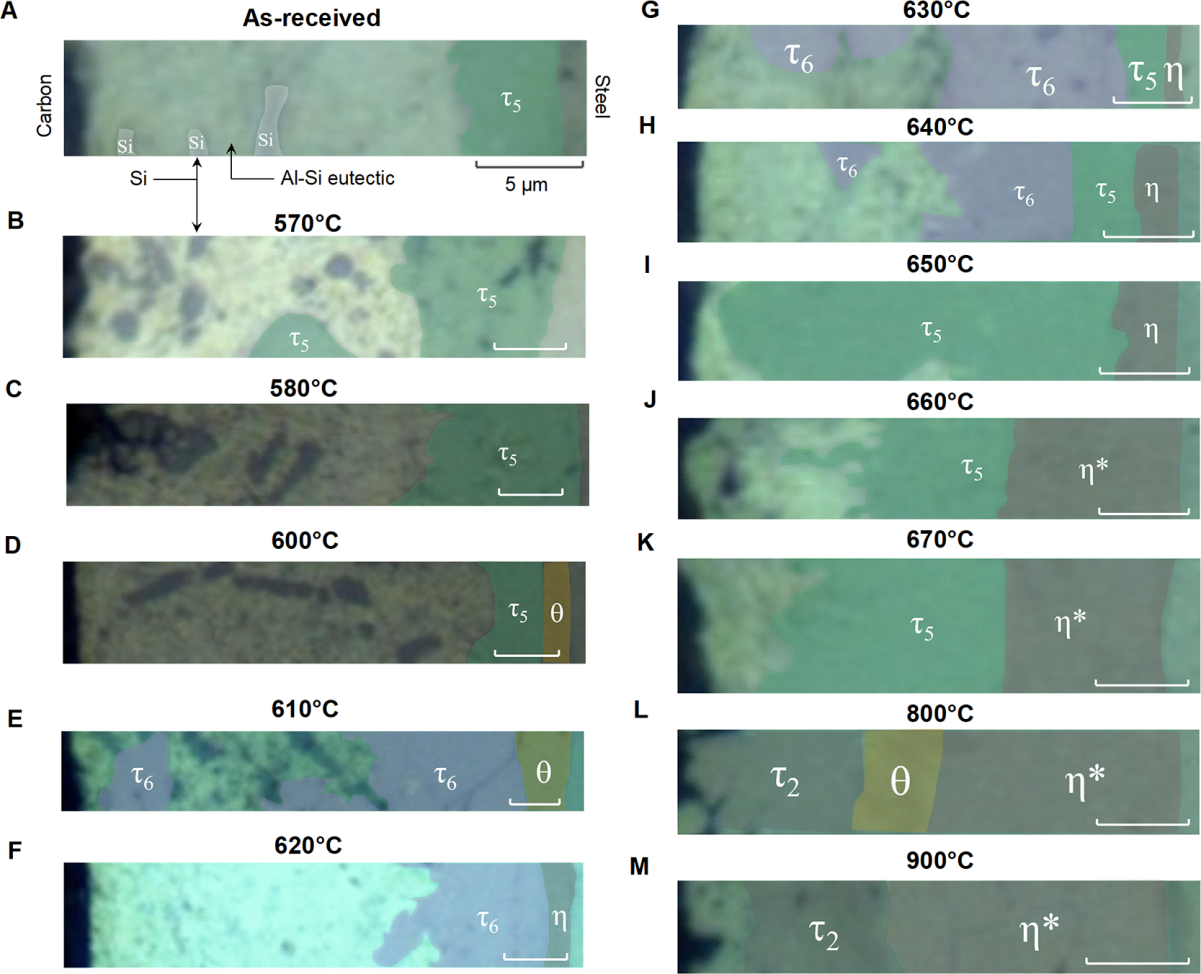
Chemical maps derived from Raman microscopic mapping of Al-Si coatings on steel substrates. (A) An as-coated sample is labeled with carbon matrix, Si, Al-Si eutectic, τ_5_, and steel to show the layout of the maps. Additional maps for samples heated for 10 min at (B) 570, (C) 580, (D) 600, (E) 610, (F) 620, (G) 630, (H) 640, (I) 650, (J) 660, (K) 670, (L) 800, and (M) 900 °C have the similar layout with the as-received sample. The scale bar in each panel corresponds to 5 μm.

Cross-sectional Raman microscopic mapping enables identification of the temperature thresholds and location at which major chemical reactions take place during structural evolution of the Al-Si coatings. As-received samples have a single layer of τ_5_ on top of the steel substrate, beneath a layer of Al-Si containing elemental Si particles (Figure 5A).^9^ It is well established that the Al-Si mixture melts at 577 °C and has been demonstrated for Al-Si-coated 22MnB5 steel.^5,9,18,25^ Raman maps indicate that the structure and composition of the coating remain stable up to 580 °C (Figure 5B,C), despite the melting of the Al-Si overlayer. A chemical reaction then begins at approximately 600 °C, with a thin layer of θ forming at the interface between τ_5_ and the steel substrate (Figure 5D). At 610 °C, the θ layer remains approximately static, while the intermetallic layer on top of it grows and transforms from τ_5_ into τ_6_ (Figure 5E). Heating to 620 °C converts the θ layer to η (Figure 5F), leading to the subsequent formation of τ_5_ at the interface between η and τ_6_ (Figure 5G). The thickness of the τ_5_ and η layers begins to increase at 640 °C (Figure 5H). Full conversion of τ_6_ into τ_5_ is achieved at 650 °C, with concomitant growth of the τ_5_ layer (Figure 5I). The Raman spectra for η transform to that designated as η* at 660 °C, with the layer significantly increasing in thickness. This η* layer then persists above 670 °C (Figure 5J,K). The greater intensity and better-defined peak shapes in the Raman spectra (Figure 2) suggest that this transformation results in a higher degree of crystallinity. Growth of θ at the interface between η* and τ_5_ is then observed at 800 °C, along with the formation of τ_2_ atop the θ layer (Figure 5L). Transformation of θ to τ_2_ at 900 °C (Figure 5M) suggests that a τ_5_ to θ transformation precedes a θ to τ_2_.

## Discussion

Raman microscopic mapping shows the emergence of intermetallic compounds at temperatures that are consistent with observations in previous studies. In the case of the as-received steel, for example, the dip-coating process is known to produce a layer of τ_5_ at the steel interface.^4,8,10,43,44^ This layer acts as a barrier between the Al-Si coating and the Fe in the substrate steel, thereby inhibiting further transformation of the Al-Si coating up to 580 °C.^4,45^ Other compounds that have been identified as minority components after the hot-dipping process include τ_1_, τ_6_, η, and θ.^8,17,25,46^ Previous studies have reported that θ and η form at ca. 650 °C,^14^ τ_2_ at 830 °C,^8^ AlFe at 882 °C,^20^ and τ_1_ at 900 °C.^15,20^ AlFe and Al_2_Fe_2_Si emerge at higher temperatures, around 900 °C.^15,20^ Some EDS and electron back-scattering diffraction measurements suggest that Al-Si-coated steels heated to 900 °C contain mixtures of τ_1_, τ_2_, and ω (FeSi_2_),^24^ while others show τ_1_, τ_5_, AlFe, Al_2_Fe, θ, and η.^15,18,20,25,46^ Variations in the composition of the Al-Si-Fe layer may arise due to variations in heating time. The temperatures at which Raman spectra identify new phases here are consistent with these observations, attesting to the accuracy of the Raman spectroscopy-based assignment.

Spectra within the Raman maps show no contributions from common oxide compounds of silicon, aluminum, or iron. Electron microscopy has shown that such Al-Si coatings form a ca. 10 nm thick coating of Al_2_O_3_ on the outer interface that limits oxidation into the bulk of the coating.^5^ Aluminum oxide is most common in the corundum (α-Al_2_O_3_) structure, which yields eight strong peaks in the Raman spectra;^47^ iron oxides tend to form hematite at temperatures above 500 °C, which also adopts the corundum structure and exhibits seven strong Raman peaks.^48^ A total of six well-defined peaks are expected for SiO_2_.^49^ The fingerprints for these oxides are not observed in any of the spectra obtained. Any oxide coatings present are therefore believed to be restricted to surface coatings on the scale of tens of nanometers.

Past analyses of Al-Fe-Si ternary compositions have identified at least 11 invariant reactions involving intermetallic compounds in the 573 to 900 °C temperature range.^21,24^ A sample of the low-temperature reactions includes equilibria between τ_6_ and an Al-Si mixture at 573 °C; between τ_4_ and τ_6_ at 600 °C; between τ_5_ and τ_6_ at 620 °C; and between τ_4_, τ_5_, and τ_6_ at 700 °C.^21,24,50^ The complete conversion of the Al-Si coating to an intermetallic coating (Figure 5), however, necessitates that the Fe content within the coating increases with time and temperature. The chain of reaction steps needed to achieve this overall conversion is therefore tied to a line traversing the initial Al-Si binary composition in the Al-rich corner of the ternary phase diagram to the Fe-rich corner. The observed evolution of Al-Si coatings in a layered fashion creates local reaction environments and local diffusion conditions that may result in divergence from these invariant reactions.

Five major chemical reactions appear sufficient to describe the overall macroscopic coating transformation process observed here, which align with mechanisms proposed in previous studies.^5,8^ We note that the intermetallic phases appear stable to small degrees of non-stoichiometry, as evidenced by variability in the stoichiometry for multiple crystal structures published.^5,21,51^ The five reactions include (1) precipitation of τ_5_ upon Fe contacting with the liquid Al-Si mixture; (2) removal of Si from τ_5_ to yield θ; (3a or 3b) interconversion between τ_5_ and τ_6_ by changes in Fe:Si content; (4) adjusting the Fe content to convert between relatively Fe-deficient θ and Fe-rich η; and (5) accumulation of Si to convert θ to τ_2_:
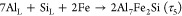
1
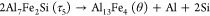
2
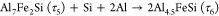
3a
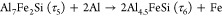
3b
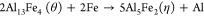
4
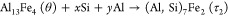
5

The consistent thickness of the τ_5_ layer up to 600 °C confirms that reaction 1 is self-limiting within the liquid Al-Si medium, implying that leaching of Fe into τ_5_ is extremely slow. Formation of θ at the internal τ_5_|steel interface requires depletion of Al and Si, as represented by reaction 2. It is notable that this region is spatially isolated from the liquid-phase Al-Si mixture and the 600 °C temperature is below any reported invariant reactions involving τ_5_.^5,8,21,24^ The excess Al and Si must therefore be transferred into the τ_5_ overlayer—this is visible with the conversion of τ_5_ to τ_6_ through reaction 3a at 610 °C. The 620 °C conversion of θ to the more Fe-rich η phase at the steel interface is attributed to reaction 4, driven by a localized increase in the rate of Fe leaching. Previous studies have demonstrated that the appearance of η is critical for growth in the thickness of the intermetallic layer,^52^ which suggests that η facilitates both dissolution of Fe from the steel substrate and diffusion into the depth of the coating. Evidence for η facilitating Fe leaching and diffusion is seen here as a reappearance of τ_5_ at 630 °C at the internal τ_6_|η interface and its subsequent growth until 650 °C. We propose that this reaction proceeds via Fe enrichment of τ_6_ through reaction 3b. Growth in the thickness of η with further temperature increase likely proceeds through a sequence of reactions 2 and 4, which would involve diffusion of Si toward the exterior of the coating and diffusion of Fe from the steel substrate to the resultant η|θ interface with paired Al diffusion toward the steel. Evidence for such a two-step process is seen in the data at 800 °C, where a layer of θ sits atop η, and the data at 900 °C, where θ is converted to the Si-rich τ_2_ phase through reaction 5. This analysis suggests that three of the phases are critical for structural evolution of the coating: τ_5_ appears to be the preferred product until the Fe content becomes too high; η is critical for facilitating the leaching of Fe from the steel substrate; and τ_2_ becomes the phase where all Si from the initial Al-Si mixture is accumulated.

The Raman microscopic mapping protocol developed here provides a rapid analysis that is complementary to traditional analyses previously employed to study the evolution of Al-Si coatings. The structural evolution of these coatings has been most frequently studied using cross-sectional SEM-EDS analysis, where morphology and elemental composition enable the distinction of different phases.^18^ This approach has a high spatial resolution, but phase identification relies upon an approach that lacks chemical specificity. Specifically, SEM provides visualization of a surface, while EDS probes elemental composition within a volume that typically spans several micrometers in three dimensions. Consequently, it is necessary to assume that the sample is homogeneous across its depth and that the volume being probed does not extend to neighboring compounds. Raman microscopic mapping sacrifices some spatial resolution, but vibrational fingerprints provide chemical specificity that boosts confidence in the identification of chemicals of interest. The dispersion of sub-micrometer particles of secondary phases throughout the coating cannot be excluded, but bulk phase transitions such as those observed here can be tracked with high confidence. The Raman microscopic mapping process developed here not only provides insight into the mechanism by which Al-Si converts to intermetallic compounds but also an accessible tool that facilitates confident phase identification. This will enable the development of strategies capable of rapidly analyzing structural evolution in such coatings, such as through combination of image segmentation and machine learning protocols to study semi-automated structure analysis. This will, for example, expedite analysis of how structural evolution mechanisms change with changing heating duration, temperature, coating formulation, or heating rate. These results provide the possibility for optimizing industrial production.

## Conclusions

The structural evolution of Al-Si coatings on a 22MnB5 steel alloy during heating was analyzed using cross-sectional Raman microscopic mapping. The Raman spectroscopic fingerprints for individual intermetallic compounds were identified by synthesizing and characterizing the compounds of interest. This information was used to identify the components present in 13 specimens that were heated to different set-point temperatures. Spectroscopic mapping demonstrated that the coating structure evolves in a layered fashion, consistent with past observations. The bulk structural evolution under our heating conditions consists of a sequence of reaction mechanism steps that occur at discrete interfaces. Analysis of the nature of these transitions leads us to propose that structural evolution is dominated by five critical reaction steps, with each step driven by gradual diffusion of Fe and Si toward the exterior interface and aluminum toward the inner interface. Important roles are observed for η, which facilitates transfer of Fe from the steel substrate to the coating and subsequent diffusion outward, and τ_2_, which is the ultimate destination of Si from the original Al-Si mixture. These insights build on past studies, which have also shown that both the rate of heating and the temperature dwell time can affect observed structures. The low cost and rapid analysis of thin coatings demonstrated here can be applied to build a comprehensive understanding of the interfacial reaction mechanisms; the technique can also be applied to quantitatively test strategies to manipulate how the structure of such Al-Si coatings evolves. It is expected that this will facilitate the development of strategies to speed up the solidification of Al-Si coatings, which would significantly decrease furnace maintenance fees in the global automotive hot-stamping industry.

## Supporting Information Available

Rietveld refinements for τ_2_, τ_3_, τ_4_, τ_5_, τ_6_, and θ and Raman spectra for τ_6_ and unique intermetallic compounds existing in different temperature-heated samples (PDF)
