# Supplementary material for: Raman Spectroscopic Analysis of the Reaction between Al-Si Coatings and Steel
Source: ACS Omega. 2023 Jul 17;8(30):27002–9. doi: 10.1021/acsomega.3c01938 (PMC10398698; doi:10.1021/acsomega.3c01938)
Supplement: Supplementary file 1 — ao3c01938_si_001.pdf [file ao3c01938_si_001.pdf]

Raman Spectroscopic Analysis of the Reaction Between Al-Si Coatings and Steel

Jixi Zhang[a], Kyle J. Daun[b,c], Rodney D. L. Smith[a,c,*]

[a]*Department of Chemistry, University of Waterloo, 200 University Avenue W., Waterloo, Ontario, Canada N2L 3G1*

[b]*Department of Mechanical and Mechatronics Engineering, University of Waterloo, 200 University Avenue W., Waterloo, Ontario, Canada N2L 3G1*

[c]*Waterloo Institute for Nanotechnology, University of Waterloo, 200 University Avenue W., Waterloo, Ontario, Canada N2L 3G1*

Correspondence to:

rodsmith@uwaterloo.ca

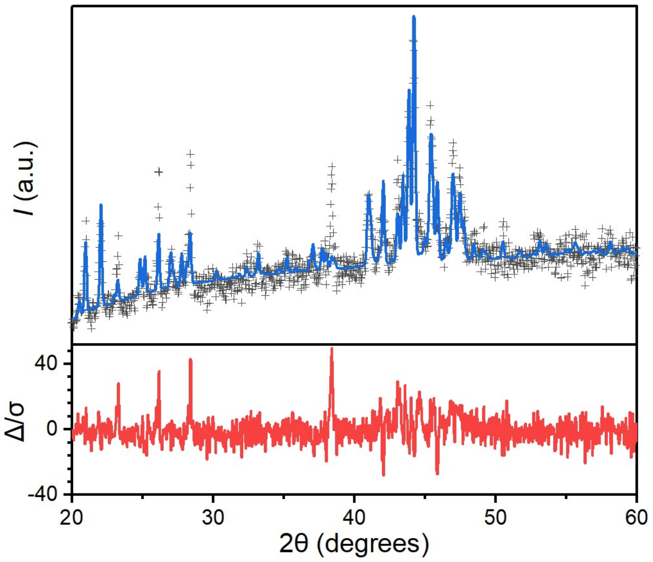

Figure S1. Rietveld refinement for synthesized τ_2 sample. Refinement performed using *R-3* space group as in ICSD 99169.

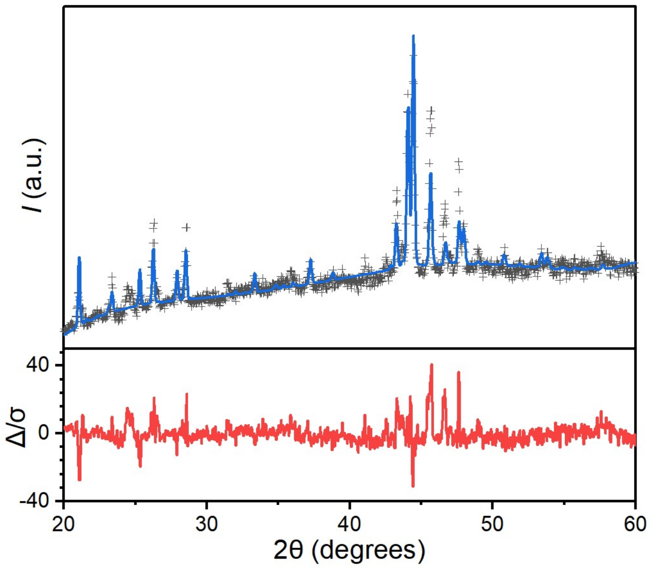

Figure S2. Rietveld refinement for synthesized τ_3 sample. Refinement performed using *Cmma* space group as in ICSD 40317.

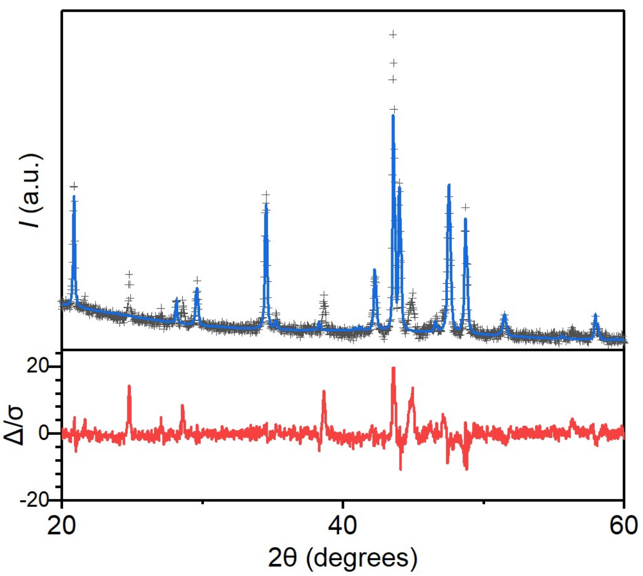

Figure S3. Rietveld refinement for synthesized τ_4 sample. Refinement performed using *I4/mcm* space group as in ICSD 199347.

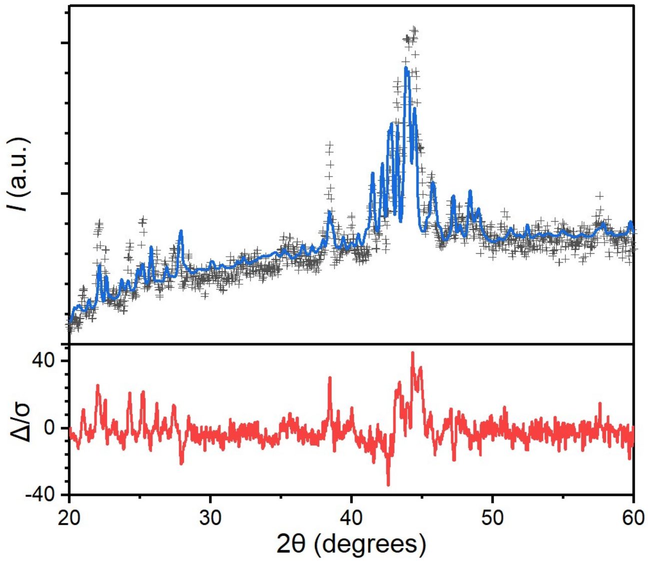

Figure S4. Rietveld refinement for synthesized τ_5 sample. Refinement performed using *$P6_3/mmc$* space group as in ICSD 42224.

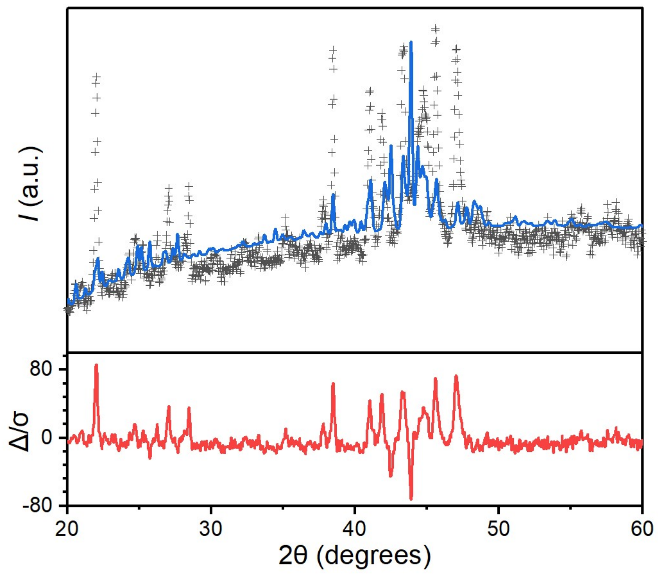

Figure S5. Rietveld refinement for synthesized τ_6 sample. Refinement performed using *A2/a* space group as in ICSD 54050.

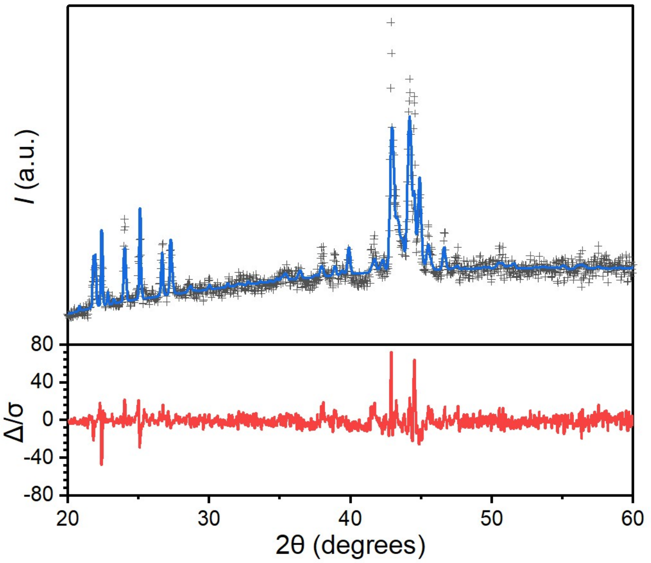

Figure S6. Rietveld refinement for synthesized θ sample. Refinement performed using *C2/m* space group as in ICSD 57795.

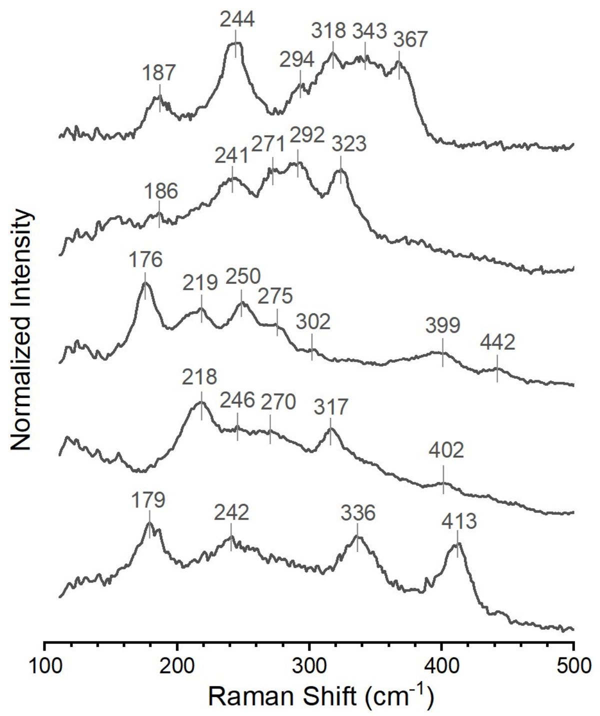

Figure S7. All unique Raman spectra observed within sample following attempted synthesis of τ_6. The upper three match τ_2, τ_4 and θ. The bottom spectrum is assigned to τ_6 and the second from bottom remains unassigned.

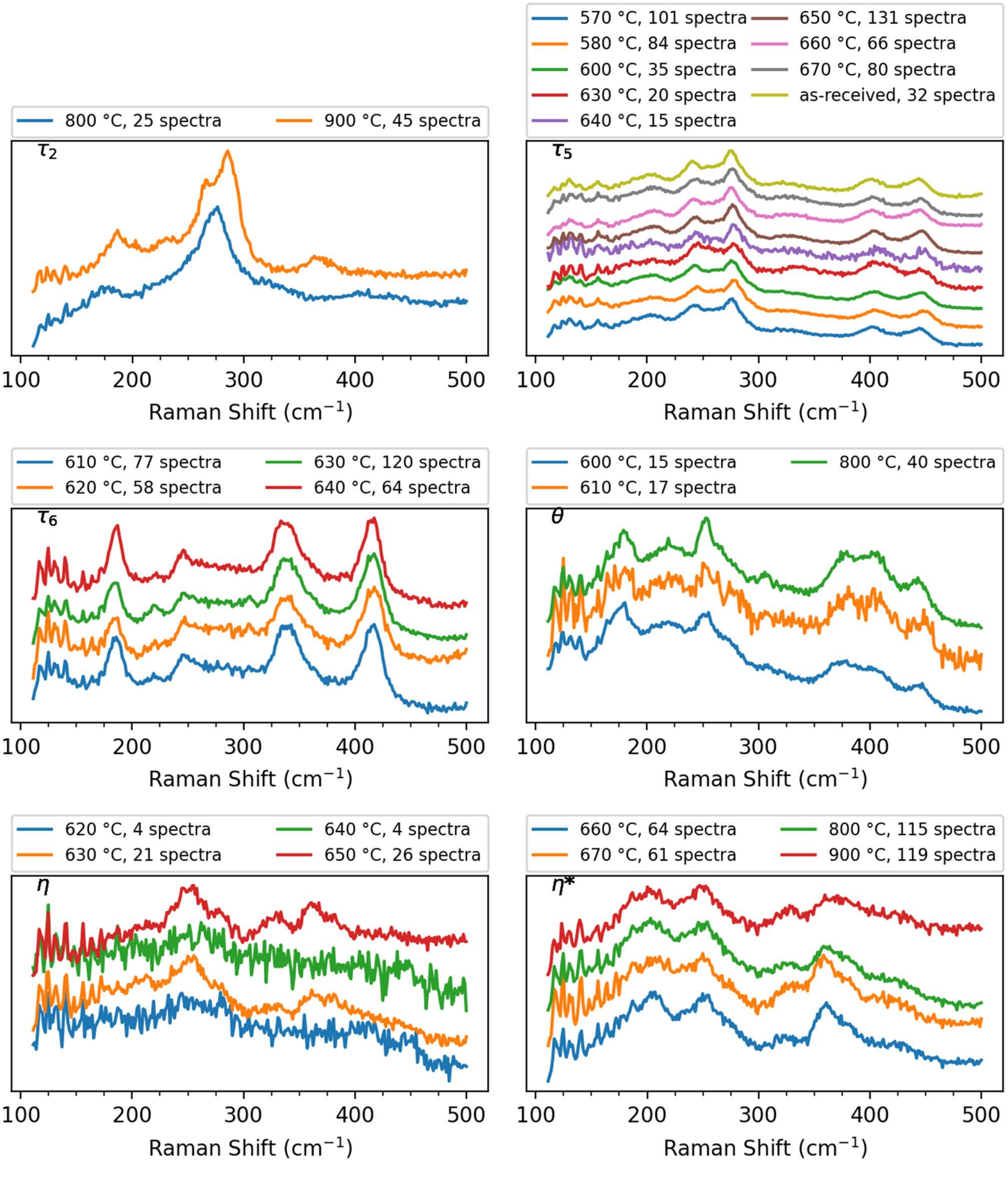

Figure S8. Average Raman spectra observed in 2-dimensional Raman microscopic maps of thin films coatings after heating. The average spectrum for each individual intermetallic compound was calculated on a per-sample basis. The number of samples involved in each calculation are noted.
